# Green communication for cognitive radio networks based on game and utility-pricing theories

**DOI:** 10.1371/journal.pone.0235953

**Published:** 2020-08-25

**Authors:** Fadhil Mukhlif, Kamarul Ariffin Bin Noordin, Omar B. Abdulghafoor, Tengku Faiz Tengku Mohmed Noor Izam

**Affiliations:** 1 Department of Electrical Engineering, Faculty of Engineering, University of Malaya, Kuala Lumpur, Malaysia; 2 Electronic and Telecommunication Department, College of Engineering, The American University of Kurdistan, Simele, Iraq; RMIT University, AUSTRALIA

## Abstract

The most crucial challenge in the functioning of the wireless networks is the efficient utilization of radio resources. A significant element of resource handling is power regulation. With increasing requirement of wireless data transmission services, it is essential to devise energy harvesting techniques for mobile devices. In this research, a new methodology has been proposed for distributed power regulation in cognitive radio, networks of CR are grounded on non-cooperation game phenomenon and pricing technique. QoS (Quality of service) of the user of CR is anticipated as a beneficial activity through pricing as well as dissemination of energy generating as an unbeneficial game wherein the consumers increase their overall efficacy. The price is defined as an actual function of transmission power to upraise the pricing of the most distant consumers. The proposed mathematical model shows that the proposed game model has a Nash equilibrium and is also unique. Furthermore, in order to make the proposed algorithm valid for green communication within the wireless network, the best response technique was proposed. Finally, simulation results showed that the proposed energy harvesting technique, grounded on a unique function of the utilization, reduces the consumption of transmission power and greatly improves the convergence speed; which are suitable for the vision of the 5G networks.

## Introduction

Going from 2G to 4G technologies, there has been an extremely fast evolution and progress of technologies particularly with respect to the computer networks and wireless networks. The primary force for this occurrence has been the demand for better energy efficiency, lower latency, and bandwidth. Thus, 4G is truly mobile broadband, even though the first standard for mobile broadband is considered to be the 3G technology. The 3G technology was originally designed for voice transmission, for the transmission of data and multimedia, certain specific considerations were taken, whereas 2G was considered as the foremost digital voice transmission having a better range of coverage. An improvement has been seen in the rate of data transmission in 2G ranging from 64 kbps to 2 Mbps in 3G while 4G has 50–100 Mbps. It is supposed that the 5G networks will enhance the speed of data transfer along with the connectivity, efficacy of the energy and measurement of the network. It is expected that 50 billion devices will be linked to the worldwide IP network by the year 2020, that is a daring task [1, 2] and [3]. Thus, the below given are the most crucial components for describing 5G: high reliability, high throughput, energy efficiency, increased scalability and low latency [4]. Hence, users will be able to experience seamless network connectivity [5]. Therefore, this upcoming technology in the wireless networks’ domain is highly likely to incorporate a ground-breaking architecture that fulfils the demands of the next-generation networks. Consequently, Cognitive Radio Network (CRN) is one of the most significant contenders for 5G network [6, 7].

Nonetheless, Cognitive radio as an emerging technology has the ability to enhance underutilization problem of spectrum faced by existing wireless networks. The basic standard of CRN use boosts up flexibility and effectiveness in the use of spectrum by permitting the unauthorized (secondary) consumers to use the resources possessed by the authorised (primary) consumers resourcefully. Along with several performance challenges and design in carrying out the CRN, and a peaceful coexistence between the primary and the secondary users is one of the most difficult challenges [8, 9].

The imbalanced and self-managed nature of CRN creates quite complicated resource management issues such as the regulation of power. Cognitive radio can also be considered a smart software-defined radio with an additional ability to sense spectrum and make decisions wisely according to their own interest and calculated decision-makers who function according to their self-concern. Secondary users struggle with one another to increase their utilisation whereas primary users levy charges from secondary users for the consumption of resources like the power to increase their profits. Thus, pricing is an important factor with respect to the interaction between the primary users and secondary users. In CRNs, secondary users are price payers, they perform tactically taking into consideration the cost and the rivalry they come across while primary users are generating the price by utilising the imperceptible indicator to assign resources and increase their returns [10, 11]. The effective handling of the resources is necessary for wireless networks generally having limited radio spectrum, an undependable channel of propagation and user movement. A significant element of the management of radio resources is the power control [12, 13].

In CR devices, an effective technique is needed in order to decrease the interference produced along with the sharing of spectrum for controlling power. The inevitability of signal-to-interference ratio (SIR) is stabilized by power control and it also enhances battery life for CR devices. The primary conception of the game and economic theory has recently been implemented to fix the issue of power control in wireless networks using various methods, where the preference of facility is not independent of the utility function. Development of a utility function is a significant element on a particular part of game theory approach that has an influential effect, and the consequences of this approach are nontrivial [14]. Different procedures are developed as a power control method considering price and utility functions. Few researches have recommended utility based on the pricing and utility functions, so the users try to increase the utility in a selfish way. Here, the utility function characteristics could be quasi-concave and selecting an optimum point in the proximity of the practical variable range of the utility function, with maximum and minimum power can be affected by the behaviour of other users. The efficiency of energy is recognized as a particular instance of utility function wherein it impacts physically; that is, the bits number magnificently obtained per joule of energy used. Each user adjusts the transmission power to attain the requisite SIR which is indirectly described in the utility function; rather it is dependent on the function for asserted efficiency [15]. However, Authors in [15] develop an approach for distributed power control in CRN based on utility-pricing technique. The utility function is chosen as a QoS of CR user based on pricing technique and a distributed power control algorithm is developed as a non-cooperative game in which users maximize their net utility. The pricing function is defined as a real function of transmit power to increase pricing charge of the remote CR users and the existence and uniqueness of the NE has been proved analytically. However, the convergence of the proposed algorithm to the stable point, NE, is slow which is not convenient for a more realistic scenario. Price-based power control algorithm in a CRN is studied in [16]. The proposed utility function considered the throughput fairness among cognitive wireless nodes, where the SINR information for CRs is used as reference for the pricing punishment parameter setting. However, the uniqueness of the NE is ignored in this research which make the proposed algorithm is not convenient for heterogeneous network. In [17] the authors proposed an efficient power control based on non-cooperative game theory. The pricing technique is proposed to design non-linear pricing function to add some improvement to the Pareto region in the non-cooperative power control game. However, the uniqueness of the NE is ignored in the proposed algorithm. Finally, the authors in [18] proposed modified power control algorithm based on non-cooperative game theory. Furthermore, to improve the accuracy of the proposed optimization problem, the Shuffled Frog Leaping Algorithm (SFLA) is modified and adopted by including the basic ideas of Artificial Fish (AF). However, the convergence of the proposed algorithm to the NE is even slower than that algorithm proposed in [15]. Green-CR is an increasingly promising and attractive technology that can offer better spectrum utilization and efficiency and maximize energy efficiency simultaneously [19]. In green CR scenario, a CR is granted permission to access the band belong to PU as long as the following condition/s exist: the interference produced to the PU is within the acceptable range, and the CR achieves good quality of service with the objective of energy efficient maximization. In this work, a green CR under non-cooperative power game allocation is our concentration. Moreover, the recent development in theory and technology of energy-harvesting from radio frequency signals are also developed for wireless communication networks [20–22]. Furthermore, with recent developments in wireless communication, energy harvesting is also used in CRNs as a "greenery" solution [22]. Hence, the selected performance metric in this work is derived from the energy-efficiency definition as will be explained in Eq (6) and that is matched with the above definition of green CR.

Therefore, in this research, distinct from the existing algorithms, we assess the power allocation issue pertaining to distributed Cognitive Radio (CR) in a proposed green scenario, which include multiple coexisting cognitive users set at the same frequency band within a communication system. Our main interest lies in a non-cooperative method, since the future distributed multiple cognitive users’ system could be faced with certain implementation challenges, wherein these users may fail to cooperate. Thus, autonomous distributed power allocation techniques need to be considered, which are associated with a key benefit of avoiding energy consumption usually seen in centralised policies that need substantial information exchange amongst users. In such case, the primary aim would be to ensure a predefined SIR requirement pertaining to targeted users while at the same time keeping power consumption to minimum for every user with effective optimisation of transmission power allocation.

### Contributions

This study is chiefly concerned with the effect of energy harvesting on the QoS (Quality of Service). Moreover, pricing of the services in wireless networks has emerged as a useful tool for management of radio resources due to its capability to guide behaviour of the users towards a more effective operating point. For that purpose, we present a system for power control in the CRN. The primary objective of the power control techniques in the CRN is to increase the utilisation of spectrum by permitting multiple CRs to share the authorised users. When the spectrum is shared, each of the CR terminals should be functioning within the limit of interference temperature to maintain primary user QoS and the CRN. To achieve this objective, certain experts focus on the improvement of power control techniques in the low preference unlicensed network (CR) and ignore high priority network interaction (primary user). Here, the power control technique development is same as that applied in the wireless networks without affecting the CRN structure. Thus, we use the model of utility to represent the satisfaction (QoS) a user receives by using the resources. We work on the problem of power control for one CR cell in a wireless network having *N* users where every user attempt to increase its own utility. While the resultant non-cooperative power control game has a Nash Equilibrium, it is ineffective. Thus, we use the pricing function to enhance efficiency of the technique. Further, we demonstrate that there is equilibrium in the pricing based non-cooperative power control game and it is Pareto superior in comparison to the game equilibrium which has no pricing. Nonetheless, the pricing-based game is still not able to reach a socially acceptable power solution. The key contributions of this paper are summarised below:

1. We presented a utility function of utility containing a weighted exponential of the ratio of the required SIR and the expected signal, as well as pricing function containing a power function for power transmission of the CRs.
2. The pricing and utility functions are instrumental in enabling each CR to select its transmission power efficiency. The nearest CRs is directed to the base station to attain its QoS requisites at minimum price while from the base station, it directs the most distant CRs to attain the essential QoS at a higher price to moderate the intervention.
3. It was confirmed that the presence and uniqueness of the NE of the green communication model and the criteria of the chosen pricing parameters.
4. Instead of a mathematical approach to the NE, this article provides a new illustration that offers a better understanding of the concept of NE.
5. We consider the non-cooperative game as the problem and utilise the best response technique to reach NE, the recommended efficient non-cooperative energy harvest technique can be actually applied in a disseminated manner without the need for any additional information.

### Organisation

The remaining article is organised as s: Section II sheds light on the overall model system and pertinent works along with the suggested Green Game model. Section III describes a mathematically approved process for the recommended equations. Section IV elucidates the proposed algorithm. Section V presents the outcomes and the throughput performance numerically, through which green transmission algorithm insights are attained. Section VI presents the conclusion and summarizes the key observations.

## System model & game formulation

**Fig 1:**
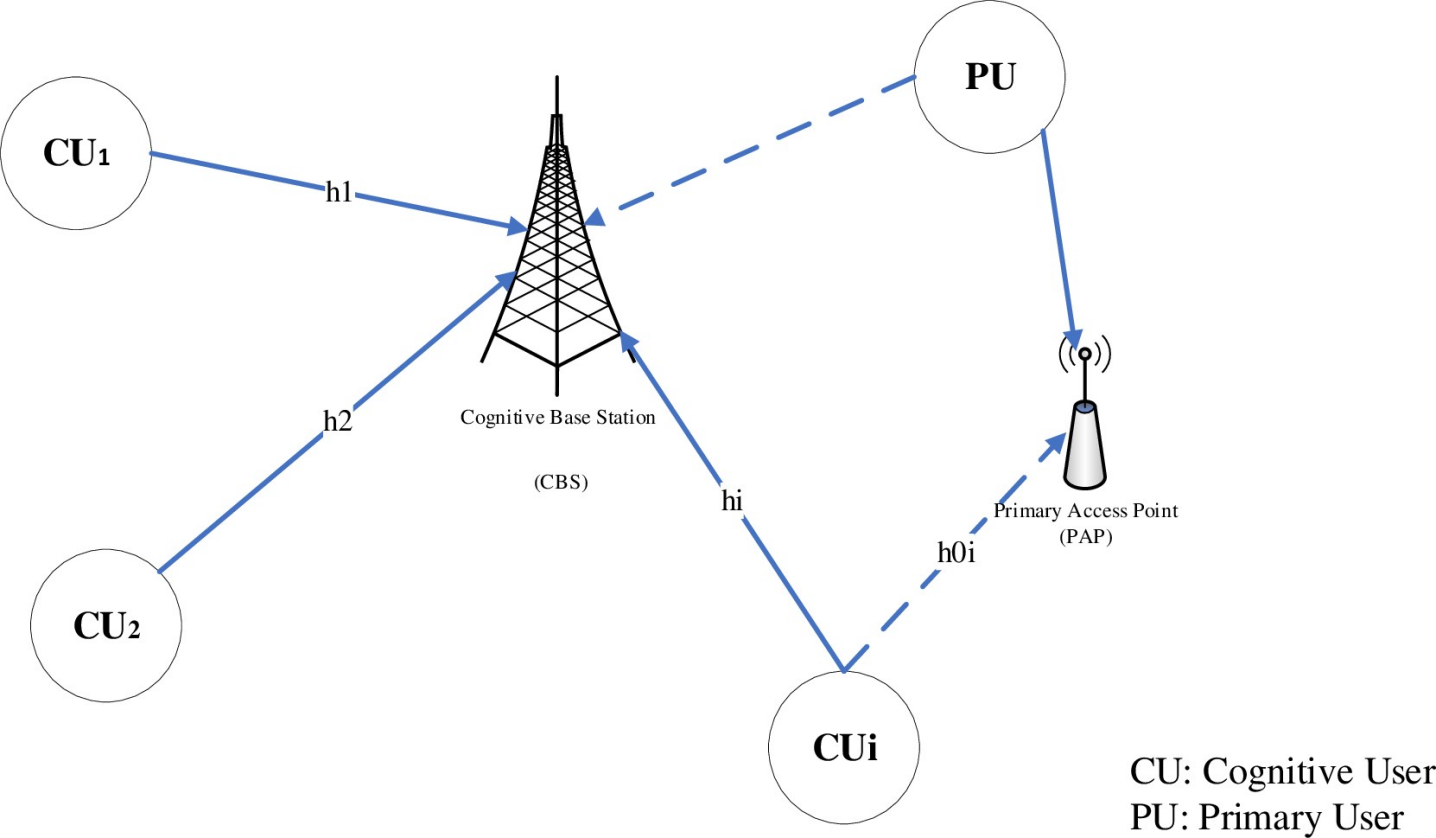
Cognitive radio network system model.

In this study, a single cell was considered including one Cognitive Radio Base Station (CRBS) coexisting with primary access point (PAP) are given in Fig 1 based on the case of the uplink power control. *N* CRs partaking of the commons licensed spectrum are connected with one primary user, and a code division multiple access (CDMA) method is applied by them to use the conventional spectrum for their transmission. It is supposed that all CRs are fixed and dispersed along with the cell’s coverage area. We denote the transmission power as *p*_*i*_ of *ith* cognitive user and the channel link gain as *h*_*i*_ between the *ith* cognitive user and CBS.

A generalised SIR formula in the single cell CRN of *ith* CR can be written as:

$$\gamma_{i}\left(p_{i}\right)=\frac{Gp_{i}h_{i}}{\sum_{j\neq i}^{N}p_{i}h_{j}+\sigma_{i}^{2}}\geq \;{г}_{i\;,i=1,2,,3,\ldots ,n}$$ (1)

Where *G* represents the spread spectrum system’s processing gain, г_*i*_ represents the threshold SIR and $\sigma_{i}^{2}$ represents the Gaussian noise power. The total amount of interference along with the noise in the denominator of Eq (1) is represented by *I*_*i*_(***p***_−*i*_), and thus Eq (1) is expressed with user transmission power function and the other user’s transmission power as follows:

$$\gamma_{i}\left(p_{i},p_{-i}\right)=\frac{Gp_{i}h_{i}}{I_{i}\left(p_{-i}\right)}=\frac{Gp_{i}h_{i}}{\sum_{j\neq i}^{N}p_{j}h_{j}+\sigma_{i}^{2}}$$ (2)

The interference based on the transmission of power by the users excepting the *ith* user is represented by the subscript −*i*.

The main goal of the system is to increase its benefit by permitting several CRs to let other users use their own spectrum. The inadequate production of the authorised users or the limit of temperature interference limits the increase in the benefit [14].

The CRs generate the overall interference power that must be below a specified limit which is known as interference temperature limit and it is expressed as below:

$$\sum_{i=1}^{N}p_{i}h_{0i}\leq I_{TL}$$ (3)

Where *h*_0*i*_ represents channel, benefit obtained by cognitive radio *i* transmission to the primary system’s access point and *I*_*TL*_ represents the limit of the interference temperature.

### Non-cooperative power control game with pricing

Game theory concepts and microeconomics are employed to describe QoS of the users concerning utility function rather than SIR [14]. Generally speaking, the model of energy harvesting game consists of three components: (i) CRs (end users) that correspond to the decision-makers or the players of the game, (ii) power strategy which corresponds to the action space or strategy of the game and (iii) user preferences (utilization function). Every player performs its function is to increase utility selfishly in the network. The NPGP (non-cooperative green communication) game model is presented based on the following formula:

$$\Phi =\left[N,\left\{P_{i}\right\},\left\{U_{i}\left(.\right)\right\}\right]$$ (4)

Where N = {1,2,…,N} represents the player’s set of indexes CRs, $P_{i}=[0,P_{i}^{max}]$ Denotes the minimum and maximum power for the *i*^th^ CR. The function of utility belonging to the user *i* is termed as *U*_*i*_(.), wherein every user belonging to the network aims to increase its efficacy in a self-centred way. To decrease consumption of power the CRs to reach the prerequisite SIR and moderate intervention in the network, the utility function of energy game harvests in Eq (4) should regard the subsequent characteristics as given by [23]:

- The utility function of CRs delivers power as SIR. The CR SIR is a function of CRs transmission as well as that of the other users.
- When there is an increase in the power used by the CR, it will also increase the SIR of that user, though it will reduce SIR of other CRs.
- To predefine SIR, the CR chooses to use low power for extended battery life and decrease interference.
- In lieu of a predefined power, the CR chooses to have greater SIR to get good channel condition.

Like any wireless network, in a CRN, every CR sends its data over the medium of air through the various systems of access. Subsequently, all the signals share air as a medium; every CR signal can be viewed as interference for the signals of the other users. Besides this interference, the fainting background and multipath noise distort the signal as it is transmitted between the source and the destination. The SIR’s denominator in Eq (1) corresponds to the signal’s characteristics. Moreover, CR users are devices that are usually battery-operated, and thus the power used for transmission is another significant factor for them. Hence, transmission power and SIR are the most significant factors that are used to express the model, which in turn determines the satisfaction of the user of the network [24].

Information sent to the destination from the source in CR and wireless data networks is represented by packets or frames of length *M* bits, having *L*<*M* bits of information with a data at the rate of *R* bits/sec. In the received data, the system identifies the errors and the erroneous data are retransferred, obtained throughput *T* can be demonstrated as:

T=Rf(γ) (5)

Where *f*(*γ*) represents the transmission efficiency function. This function *f*(*γ*) must be based on the SIR obtained through the channel, and its value ranges from 0 to 1 (i.e., *f*(*γ*)∈[0,1]). Moreover, assuming that *p*_*i*_ is the transmitted power for the *i*^th^ user. Hence, the proposed performance metric is derived from the energy-efficiency definition of [25]:

$$U_{i}\left(p_{i},p_{-i}\right)=\frac{LRf\left(\gamma_{i}\right)}{Mp_{i}}$$ (6)

The Nash Equilibrium caused by non-cooperative power control is not efficient since it does not take into consideration the cost it enforces on supplementary nodes by producing the interference. Thus, the pricing idea was employed to inspire users to utilise the network resources more effectively. The usual representation of the pricing-based non-cooperative power control game is given below:

$$\Phi^{c}=\left[N,\left\{P_{i}\right\},\left\{U_{i}^{c}\left(.\right)\right\}\right]$$ (7)

Where $U_{i}^{c}(.)$ represents the function of utility using pricing that is written as:

$$U_{i}^{c}\left(p_{i},p_{-i}\right)=U_{i}\left(p_{i},p_{-1}\right)-C_{i}\left(p_{i},p_{-1}\right)$$ (8)

Many works pondered upon the power control problem by providing different pricing and utility functions. Hence, selected works to be compared with are EH-NPGP [15], R-NPGP [16], NPG-ESIA [17] and NPG-MSFLIA [18].

### Proposed green-game model

We present a new utility function that depends on a novel sigmoid effective function as well as a function of power transmitting, power pricing function of the users. Mathematically speaking, efficient sigmoid function can be defined as follows:

$$f_{i}\left(\gamma_{i}\right)=\frac{1}{{\left(1+exp\left(1-z\;sir_{i}\right)\right)}^{{г}_{i}}}$$ (9)

**Fig 2:**
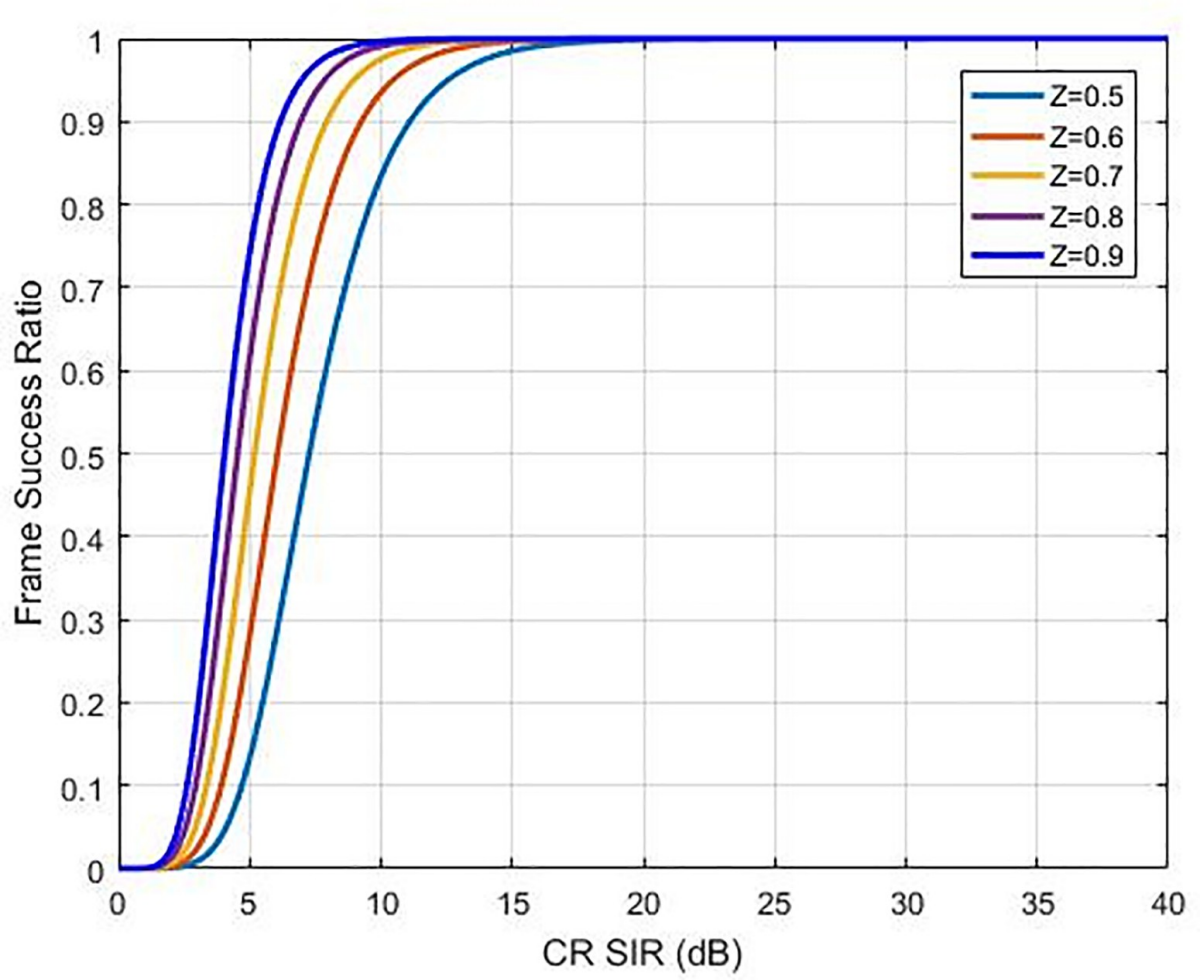
Optimal efficiency function with different z values.

**Fig 3:**
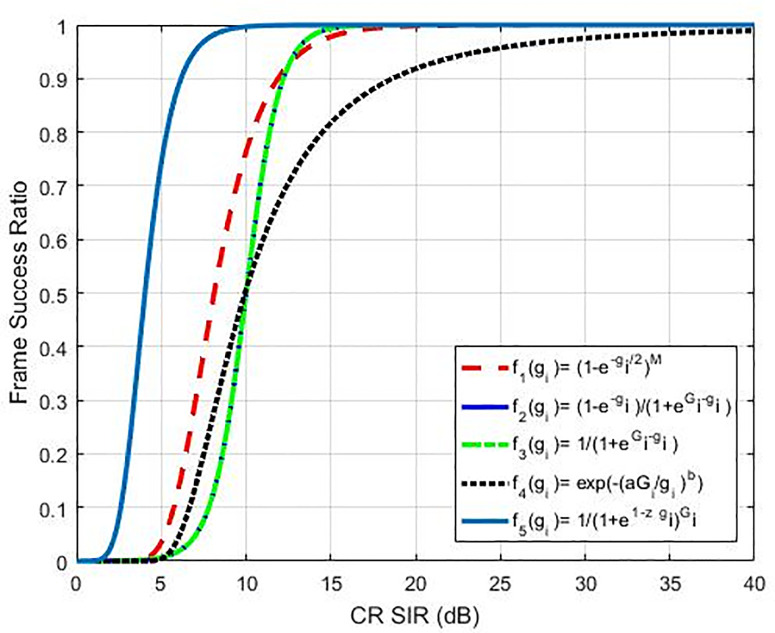
Proposed efficiency function (f_5_) compared to functions in [15], [16], [17] and [18].

**Fig 4:**
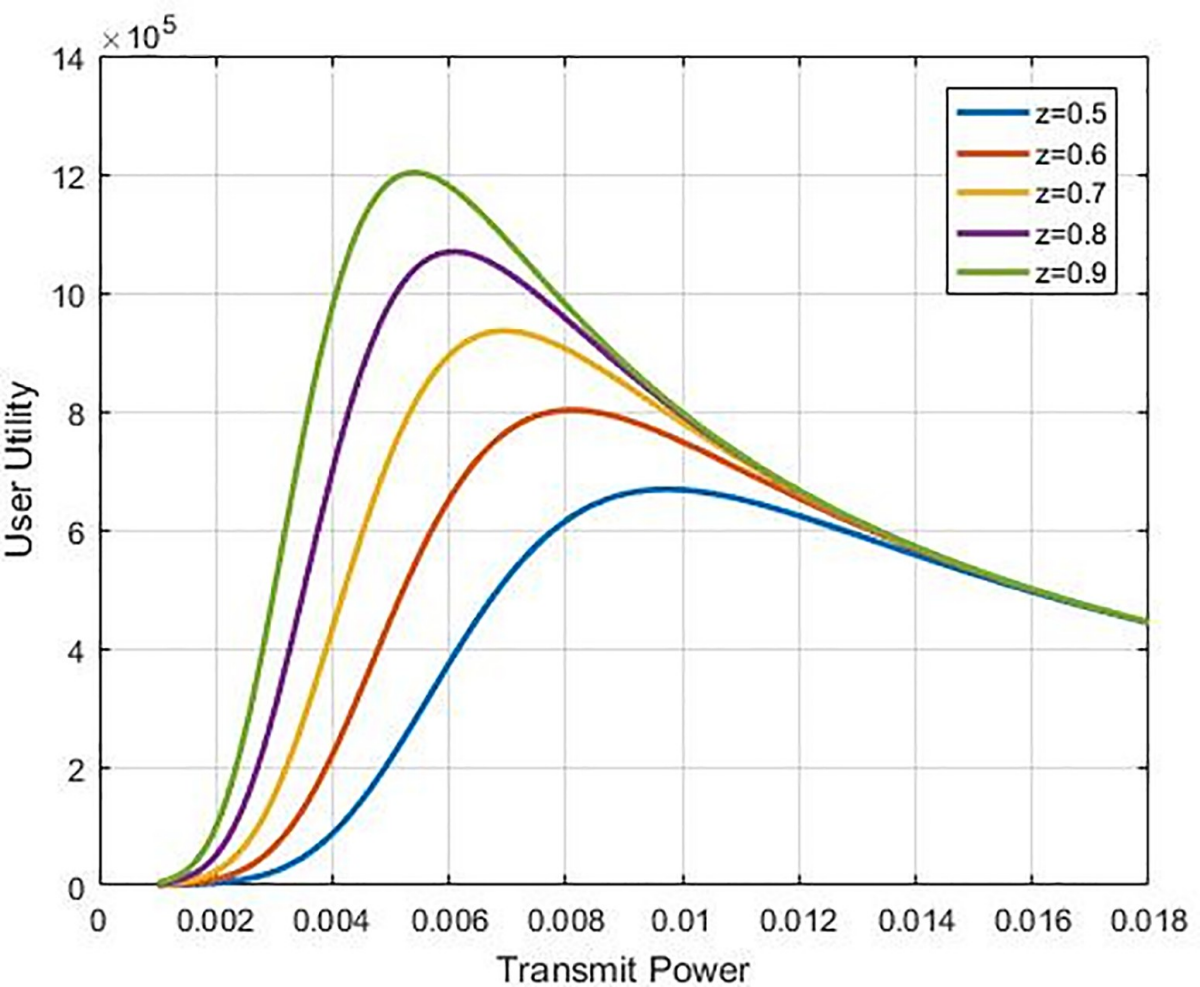
Utility function for user as a power transmission function for fixed interference and different tuning value of z factor.

Where *z* is the tuning factor where its change will change the response of the efficiency function proposed, as shown in Fig 2. With this ability in controlling response, the proposed function will be more efficient than others which are compared with as shown in Fig 3 hence, we called it an optimum function because of we could use it to control efficiency response as well controlling the utility function value as shown in Fig 4. Nonetheless, our recommended sigmoidal function is more efficient compared to others since it is simpler and more effective to handle only a single equation and regulate the efficiency function response. The above efficiency functions are compared in Fig 3. As per Eq (6), the function of utility of the *i*^th^ CR is expressed as:

$$U_{i}=\frac{LR}{Mp_{i}}\frac{1}{{\left(1+exp\left(1-z\;sir_{i}\right)\right)}^{{г}_{i}}}\frac{bit}{joule}$$ (10)

**Fig 5:**
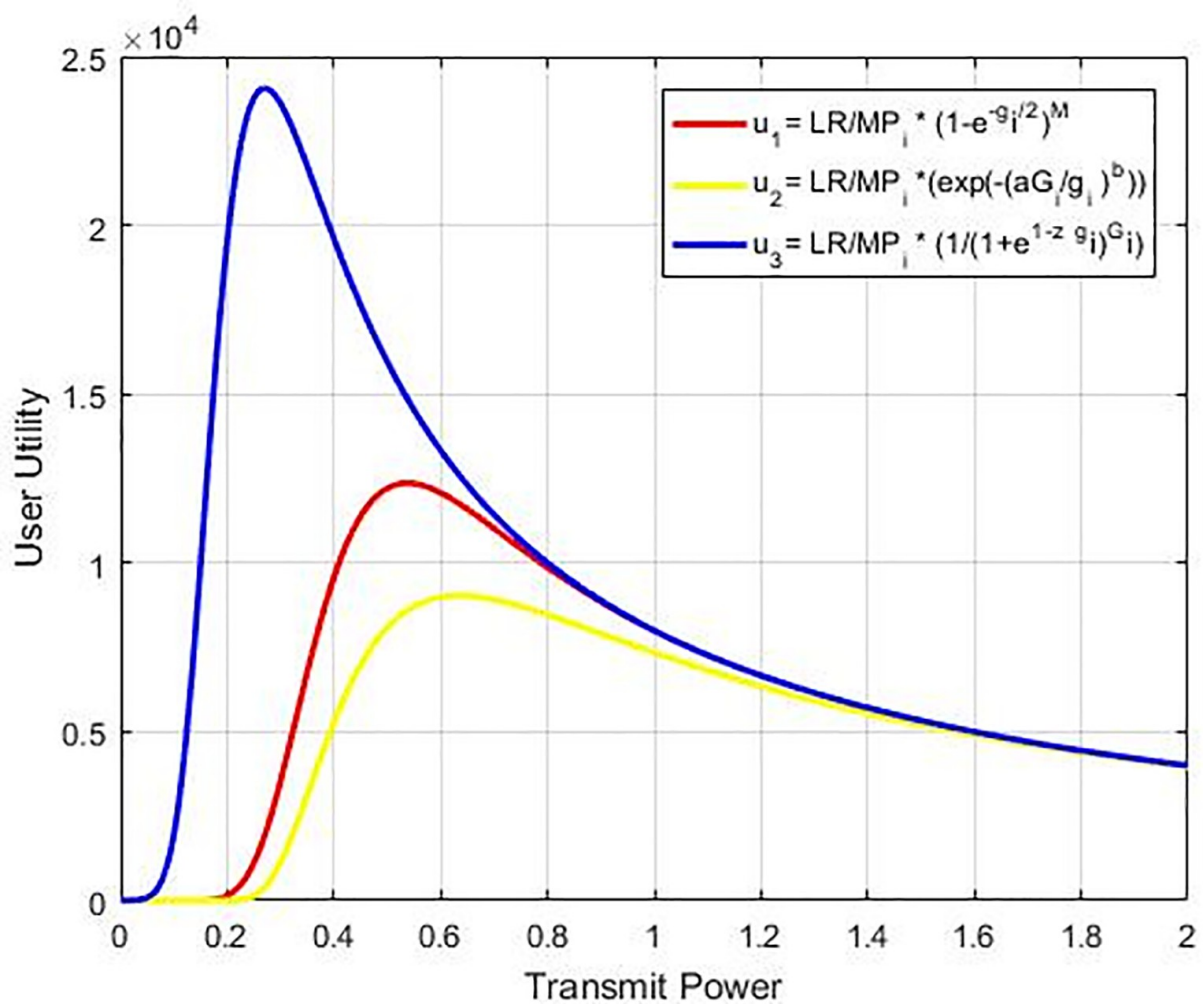
Proposed utility u3 compared to u1 [25] and u2 [15].

The utility function is given in Eq (10) corresponds to balance between the life of battery and throughput, and it is especially suitable for applications in which power saving is crucial as compared to get a better throughput like green cognitive radio [26]. We assume that the defined SIR value is determined for the cognitive radio. The adjustment of the suggested function of utility can be made by applying the factor for tuning (*z*). The user’s maximum power transmission would be altered according to the optimal function of utility. Fig 4 displays the curves of the recommended utility function regarding transmission power for various *z* values, which are presented in Fig 5. It reflects how the factor of tuning can make the recommended utility function more effective as compared to the other methods found in the literature. It can be proved that there is an increase in the utilisation and the transmission power reduces by diminishing parameter *z* value, therefore, it will reduce the system of the target SIR. The main system relays the *z* factor to CRN to tune the target SIR, based on the interference amount. A lower value of *z* is transmitted by the main system, as the interference amount almost touches the limit of the interference temperature.

**Fig 6:**
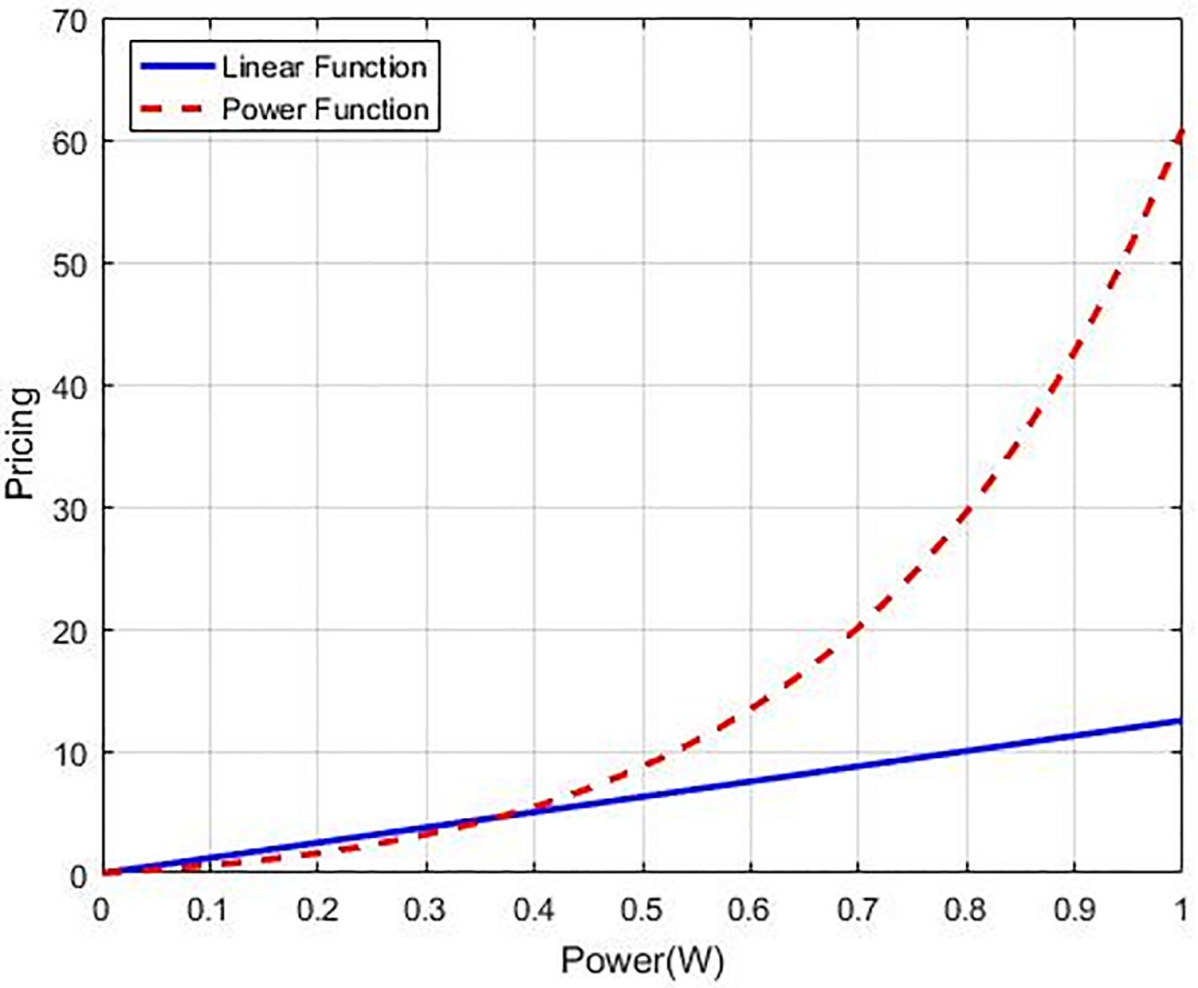
Comparison of linear and power function pricing.

Moreover, we present a new structure for pricing to improve the system’s efficiency of the system by promoting CRs to utilise the system’s resources effectively. This design contributes by applying a higher cost for users who are farthest from the base station and use more power. Thus, we present an exponential power function of the transmission power rather than the conventional linear pricing. Fig 6 displays an instance of the disparity among the power pricing and the linear pricing methods. We supposed that the power transmitted by different users ranges from minimum to maximum limit for power strategy [0,1], and a numeric calculation is made for the price functions. It can be seen that the pricing obtained by using the power function is lesser than that of the linear pricing CRs function using low transmission power and who are closer to the network while it is higher for the farthest CRs which use high power.

Hence, the suggested pricing function is stated as:

$$C_{i}\left(p_{i},p_{-i}\right)=c\;p_{i}\;exp\left(p_{i}\;\alpha \right)$$ (11)

Where c and *α* represent the pricing factor. Therefore, the pricing-based utility function is written as:

$$U_{i}^{C}\left(p_{i},p_{-i}\right)=\frac{LR}{Mp_{i}}\frac{1}{{\left(1+exp\left(1-z\;sir_{i}\right)\right)}^{{г}_{i}}}-c\;p_{i}\;exp\left(p_{i}\;\alpha \right)$$ (12)

Thus, green non-cooperative power control with pricing is the recommended in the game and described as:

$$GC-NPGP:\underset{pi\in Pi}{\max}U_{i}^{C}\left(p_{i},p_{-i}\right)=\frac{LR}{Mp_{i}}\frac{1}{{\left(1+\exp\left(1-z\;sir_{i}\right)\right)}^{{г}_{i}}}-c\;p_{i}\;\exp\left(p_{i}\;\alpha \right)$$ (13)

The benefit of a function of pricing is its capability to direct CRs towards Nash Equilibrium mark effectively. It is accomplished by enhancing pricing of the most distant users, utilising greater power transmission to communicate. Also, the function of pricing decreased the pricing used to the closest CRs who utilise lower power transmission to communicate. Every CR performs a search to increase its financial gains by regulating its power transmission distribution. Likely, the power control game causes Nash Equilibrium, and it signifies the composed power of all CRs such as not a single CR can raise the advantage by altering its power transmission. To formulate the technique used in the game of non-cooperative power control, we employ a power control method wherein every CR tries to maximise its net utilisation $U_{i}^{C}(p_{i},p_{-i})$. For the optimisation of power, the maximisation can be attained at a level where the partial derivative of $U_{i}^{C}(p_{i},p_{-i})$ corresponding to power *p*_*i*_ is equal to zero.

## Nash equilibrium in GC-NPGP

In this portion, a mathematical representation associated with the uniqueness and existence is given bellow [27]:

***Definition 3*.*1***: Nash equilibrium in the GC-NPGP method can be described as the power vector, e.g. *P*_*i*_ = [*p*_*i*_,….,*p*_*i*_], in which no participant can enhance the utility function, *U*_*i*_(*p*_*i*_,*p*_−*i*_), by independently changing its own strategy outline, i.e., *p*_*i*_. In mathematical terms, Nash equilibrium is presented as follows:

$$U_{i}\left(p_{i},p_{-i}\right)\geq U_{j}\left(p_{i},p_{-i}\right),\;⩝p_{i}\in \hat{P_{i,}}\;⩝i\in N$$ (14)

### Nash equilibrium existence

The Nash equilibrium in the recommended method provides a consistent and predictable outcome where several CRs with opposing interests participate and get to a point where no participant can request to adjust its own strategy outline. To confirm NE’s existence, the below theorem is proposed:

***Theorem 3*.*1*:** The Nash equilibrium is present in GC-NPGP = [*N*,{*P*_*i*_},{*U*_*i*_(.)}], if it fulfils the following criteria ⩝*i* ∈*N*:

1. The profile action strategy (i.e., *p*_*i*_) is a compact, convex and nonempty subset.
2. The function of utility *U*_*i*_(*p*_*i*_,***p***_−***i***_) is a concave and continuous function over strategy set of the players.

***Proof***: It can be obtained by proving that both the criteria provided in ***Theorem 3*.*1*** are satisfied by GC-NPGP. It is corroborated by the following evidence:

1. As every CR user uses a strategy outline well-defined by optimal and minimal power as given in Eq (9), the first condition is promptly satisfied.
2. For proving that the second criterion is also satisfied, the given utility function based on variable pricing must be shown to be concave in *p*_*i*_,⩝*i*∈*N*.

***Definition 3*.*2***: According to [28] Super Modular ***definition 5***, The utility function *U*_*i*_(*p*_*i*_,***p***_−***i***_) characterised by the convex set $\hat{P_{i}}$ is concave in *P*_*i*_ only in case, the surplus function’s second derivative is greater than 0 [28, 29].

To show this condition is true, the following set of Equations: $\frac{\partial^{2}u_{i}^{p}}{\partial^{2}p_{i}^{c}}>0$, must be solved ⩝*i*. Hence, the following Lemma must be satisfied.

***Lemma 3*.*1***: The utility function based on pricing provided in Eq (12) is concave in *p*_*i*_,⩝*i*∈*N*.

Considering that both the criteria are given in **Theorem 3.1** are satisfied, the recommended GC-NPGP is a concave n-player game having one or more NE in it.

### Nash equilibrium uniqueness

The strategy outline of the players carries the recommender concave and continuous utility function. Therefore, NE is present in GC-NPGP. Nonetheless, a question may arise at this juncture naturally: Is the existence of the NE unique? The uniqueness of NE can be examined as follows:

***Definition 3*.*3***: An alternative NE definition is the best response strategy which can be defined as per the following:

$$BR\left(p_{-i}\right)=\left\{p_{i}^{c}\in {\hat{P}}_{i}:u_{i}^{c}\left(p_{i}^{c},p_{-i}^{c}\right)\geq u_{i}^{c}\left({\vec{p}}_{i}^{c},p_{-i}^{c}\right),\;⩝{\vec{p}}_{i}^{c}\in {\hat{P}}_{i}\right\}$$ (15)

Also, the best strategy for response is a set including just a single maximum point that increases the objective function, which is mathematically formulated as follows:

$$p_{i=arg\underset{p_{i}\in P_{i}}{\max}U_{i}^{c}\left(p_{i},p_{-i})\right)}$$ (16)

Furthermore, the second derivative has been proven to be greater than zero, which means that the maximum point is the optimal unique point.

***Theorem 3*.*2***: The NE of the GC-NPGP game is [*N*,{*P*_*i*_},{*U*_*i*_(.)}] which is unique.

***Proof***: The main feature of the uniqueness of NE is to prove that a typical function is the best strategy for response. For the recommended game, GC-NPGP = [*N*,{*P*_*i*_},{*U*_*i*_(.)}], which is the best response given by the *i*^*th*^ user with respect to others’ power strategy.

To prove the uniqueness of NE, the function for the most suitable response must be a regular function and must also possess the following characteristics [30]:

1. Positivity: *BR*(***p***_−*i*_)>0.
2. Monotonicity: given $p\geq \hat{p}$, then $BR(p_{-i})\geq BR({\tilde{p}}_{-i})$.
3. Scalability: given, for all *ε*>1, then *εBR*(***p***_−*i*_)>*BR*(*ε****p***_−*i*_).

Moreover, it has been confirmed in [31] that if a fixed point from Eq (13) fulfils the characteristics as mentioned earlier, then the *BR*(P_*-i*_) proceeds towards a fixed point. Thus, according to [31], a fixed point in Eq (13) meets the positivity, monotonicity and scalability under the specific circumstances mentioned in theorem 3.1.

In conclusion, a standard function must be used as the best response strategy function. Thus, the non-cooperative power control system recommended by us has only a single unique NE solution which satisfies the evidence of the distinctiveness of the Nash Equilibrium.

## GC-NPGP algorithm

We propose an iterative best response technique to control all users’ transmission powers to achieve the required SIR for all CRs and ensure NE opportunistically with available information of SIR.

Nonetheless, we assume that every CR updates its transmission power at time instances *t*_*i*_ = {*t*_*i*1_,*t*_*i*2_,….}, where *t*_*ik*_<*t*_*i*(*k*+1)_, and we suppose the power strategy set of the *i*th CR is $P_{i}=[P_{i}^{min},P_{i}^{max}]$. We fix an infinitesimally small quantity *ε* where (*ε*>0) and the recommended technique is given in Eq (13) produces a sequence of powers as given below:

  GC-NPGP

    I. Initialize transmit power vector $p=[p_{1}^{0},p_{2}^{0},p_{3}^{0},\ldots ,p_{N}^{0}]$ randomly at time *t*_*0*_

II. For all *i*∈*N* at time instant *t*_*k*_;

            a) Update *γ*_*i*_(*t*_*k*_) using Eq (1)

            b) Consider the best response of power strategy *r*_*i*_(*t*_*k*_);

                    Based on $r_{i}(t_{k})=arg\underset{p_{i}\in P_{i}}{\max}u_{i}^{C}(p_{i},p_{-i}(t_{k-1}))$

            c) Allocate the transmit power as $p_{i}(t_{k})=min(r_{i}(t_{k}),p_{i}^{max})$

III. If ‖*p*(*t*_*k*_)−*p*(*t*_*k*−1_)‖≤*ε*; stop iteration and state Nash equilibrium as *p*(*t*_*k*_)

  IV. Else;

          *k* = *k*+1 and refer to step II

    V. End

**Fig 7:**
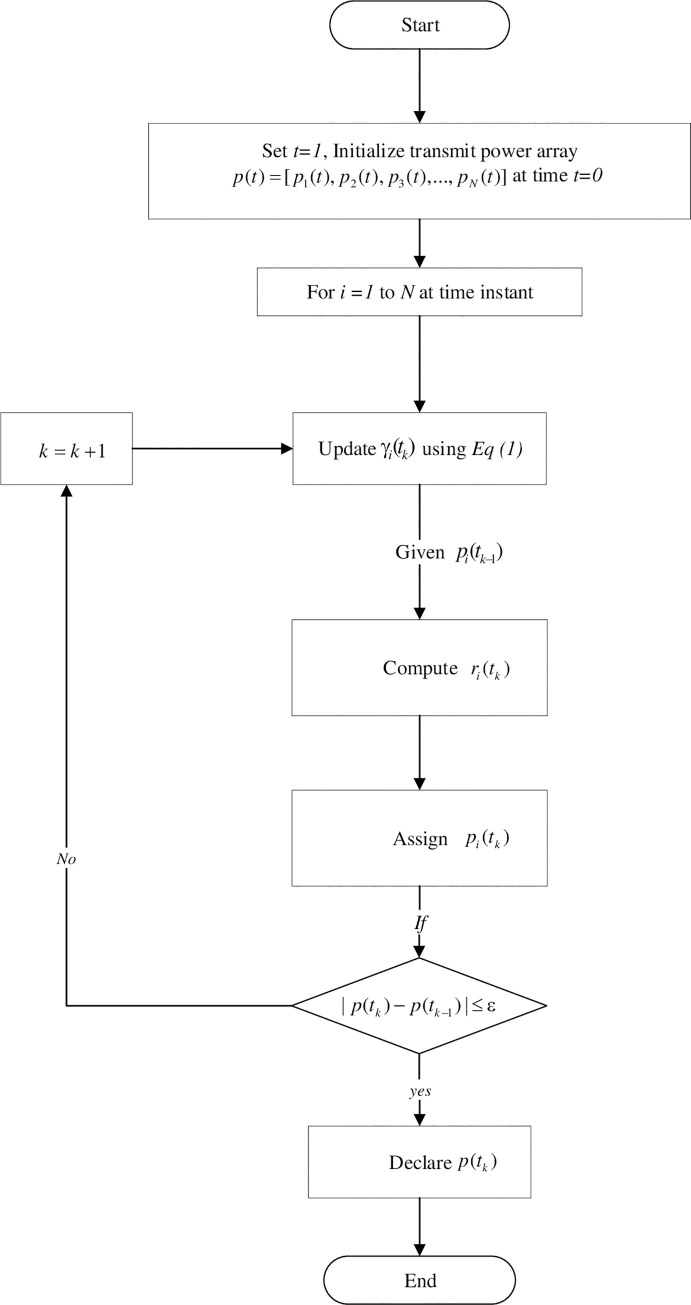
Proposed algorithm flowchart.

Where *r*_*i*_(*t*_*k*_) is the set of the most suitable transmission powers for *i*th CR which is obtained by applying objective function with constraint in best response technique at time instant *k* concerning the interference vector *p*_−*i*_(*t*_*k*−1_). It is noteworthy that the *i*th CR optimises the overall utilisation over the power strategy domain of the GC-NPGP. The presented technique determines the transmission power of the *i*th CR by choosing the smallest power among all probabilities suggested by the method. The technique will solve separately the maximum of every CRs objective. The flowchart of the recommended technique is given in Fig 7, which is giving a clear image about the algorithm with its main five steps explained above in GC-NPGP. Moreover, the proposed algorithm is based on power allocation using pricing function. Hence, the computational complexity is directly depending on the number of users and the available channels which resulted in *O*(log(*N*)).

## Numerical results & discussion

This section evaluates the functioning of the recommended power control game based best response method by us against the EH-NPGP [15], R-NPGP [16], NPG-ESIA [17], NPG-MSFLIA [18]. The same numerical calculations were employed to achieve the Nash Equilibrium solution utilities functions to reap the benefits of the recommended function of utility. The stable system variables applied in the experiment are enumerated in the below-given Table 1.

**Table 1:** System parameters.

Parameter	Value
Number of players	7
Overall number of bits per frame, M	80
Total counts of information bits for each frame, L	64
Spread Spectrum processing gain, G	70
Data rate, R	10 kbps
AWGN power at receiver, *σ*^2^	1e-14 Watts
Maximum power constraint, $P_{i}^{max}$	1 Watts
Target SIR, г_*i*_	9
Pricing factors, *c*&*α*	1e4, 2.5
Tuning factor, *z*	0.5–0.9

We regard a model for the system depends on a sole cell CRS (cognitive radio system) using a predefined packet size and without coding to forward error correction. In case of the all-purpose efficacy of function described, the SIR equilibrium is determined by resolving the formula *f*(*γ*)*γ*−*f*(*γ*) = 0 that ensures optimal utilisation of γ* = 12.4. The quantity of γ* is the actual target SIR that each of the CRs attain to maximise the efficiency of their particular utility function. The feasibility condition for γ*, for the cognitive radio system, is given by the subsequent tied up the amount of users [30]:

$$N\leq 1+\left(\frac{G}{\gamma^{*}}\right)=7\;CR\;terminals$$ (17)

As per (17), we suppose that the system has at the most 7 CRs and, their distance from the base station is *d* = [368,490,580,630,720,810,950] *m*.

In this study, we employ an uncomplicated propagation model wherein the entire route achievements determine the functions having path loss exponent (denoted by *β*), of the gap between the CBS (cognitive base station) and cognitive radio *i* and cognitive base station (CBS) and is given as follows:

$$h_{i}=\frac{K}{d_{i}^{\beta }}$$ (18)

Where the distance between base station and the *i*th user is represented by *d*_*i*_, the path loss exponent is represented by *β*, assumed to be 2 and generally from 2 to 6, and *K* = 0.097 is a persistent. This *K* value is chosen to form a transmission power of 1W for a CR terminal functioning at 950m from CBS in the system having 7 CRs and each one of them operating with γ*. Nonetheless, the current experiment shows all the cognitive users begin with preliminary power $p_{i}^{\left(0\right)}=2.22*10^{-16}\;w$ and *ε* = 10^−18^.

**Fig 8:**
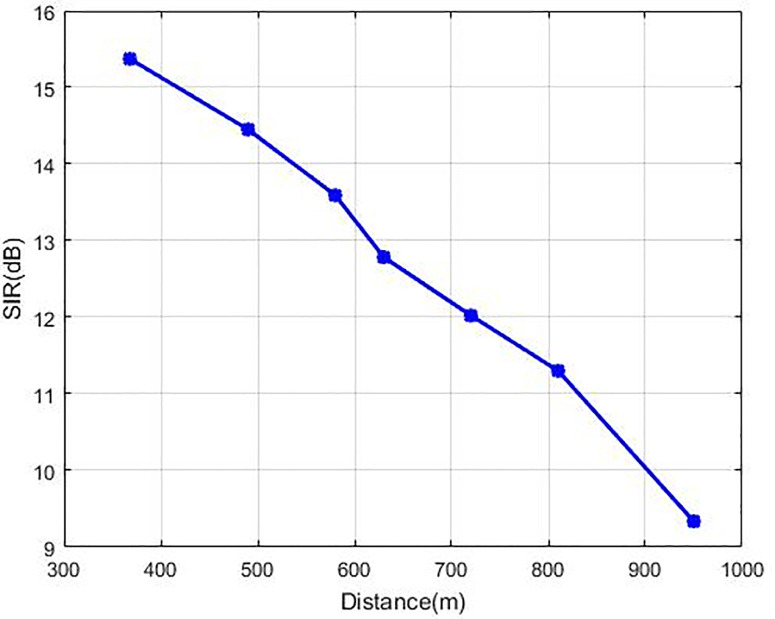
SIR Vs. distance for each user.

**Fig 9:**
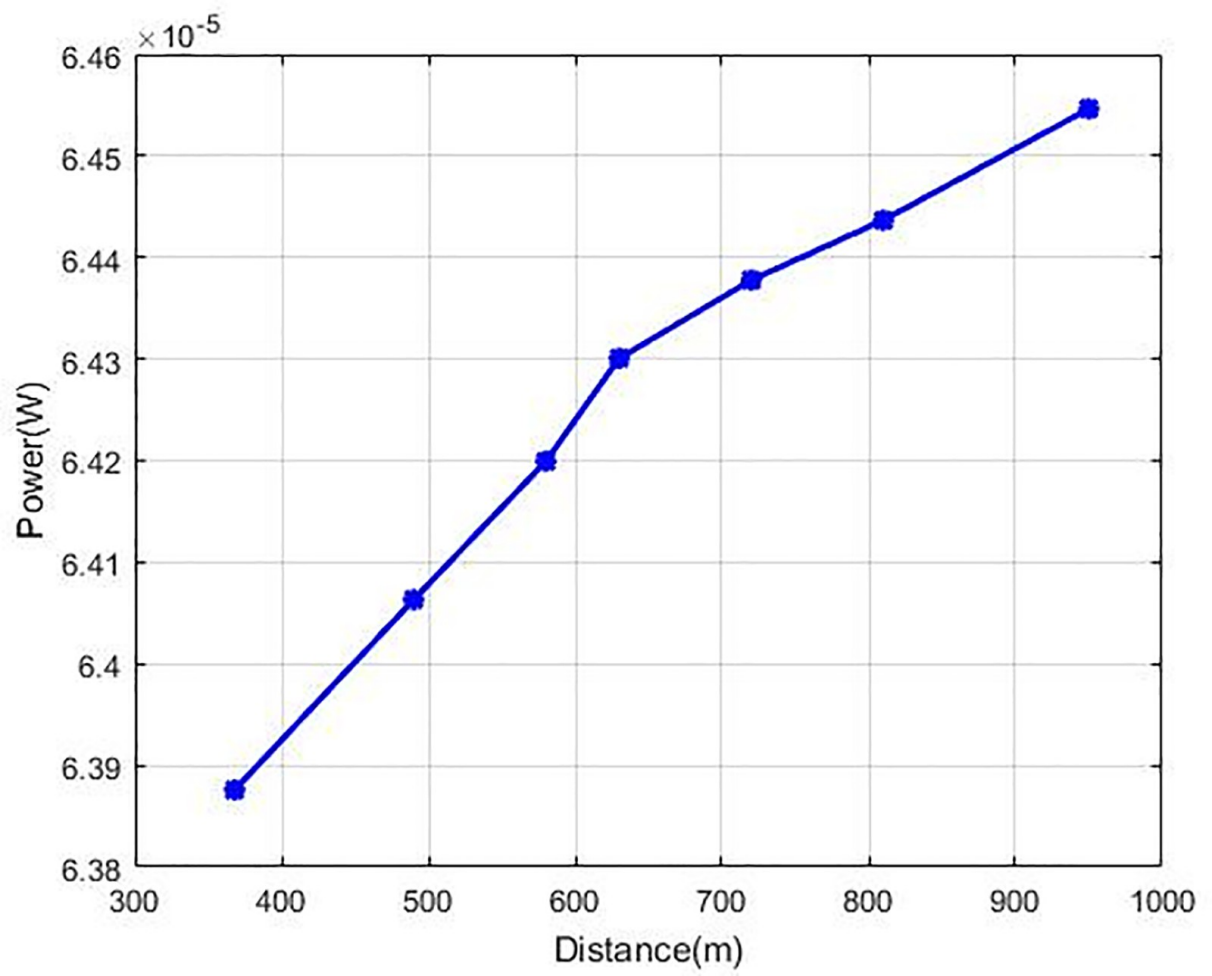
Power vs. distance for every user.

The outcomes of SIR at Nash Equilibrium are described in Fig 8. CR obtains these outcomes in accordance with the remoteness between the base station and every CR. All the users of CR s retain their SIR more than the required value (г_*i*_ = 9) and SIR value is reduced by enhancing the distance for each of the cognitive users. The Fig 8 curve of SIR demonstrates that our recommended technique GC-NPGP is more effective when there are the highest SIR values as compared to the other techniques. The highest powers are utilised by the farthermost CRs and signify the interference source, and thus our recommended technique used a greater cost to the most distant users. Fig 9 demonstrates the graph of transmission power (in Watts) against the distance of CR from the base station for the suggested method in which the transmission power increases steadily when the distance of the user is increased. Here, it is apparent that the curve of the transmission power of the recommended GC-NPGP is increasing steadily.

Table 2 demonstrates the CR users’ SIR in the last part of the network algorithm experiment. The table demonstrates that the GC-NPGP technique along with the suggested price function attain the highest SIR value in comparison to the other methods found in the literature. The SIR of the 2 most recent CR users is smaller due to application of higher cost. Thus, it can meet a better point of equilibrium by limiting the least SIR needed for terminals with poor channel conditions.

**Table 2:** Final SIR of CR users.

CR user	Final SIR of NPG-MSFLIA [18]	Final SIR of NPG-ESIA [17]	Final SIR of R-NPGP [16]	Final SIR of EF-NPGP [15]	Final SIR of Proposed GC-NPGP
1	12.41	12.42	12.42	12.69	15.33
2	12.4	12.43	12.43	12.69	14.41
3	12.4	12.43	12.43	12.69	13.56
4	12.39	12.43	12.43	12.69	12.76
5	12.37	12.42	12.42	12.68	12.00
6	12.33	12.4	12.4	12.66	11.28
7	12.26	12.3	12.3	12.4	9.32

**Fig 10:**
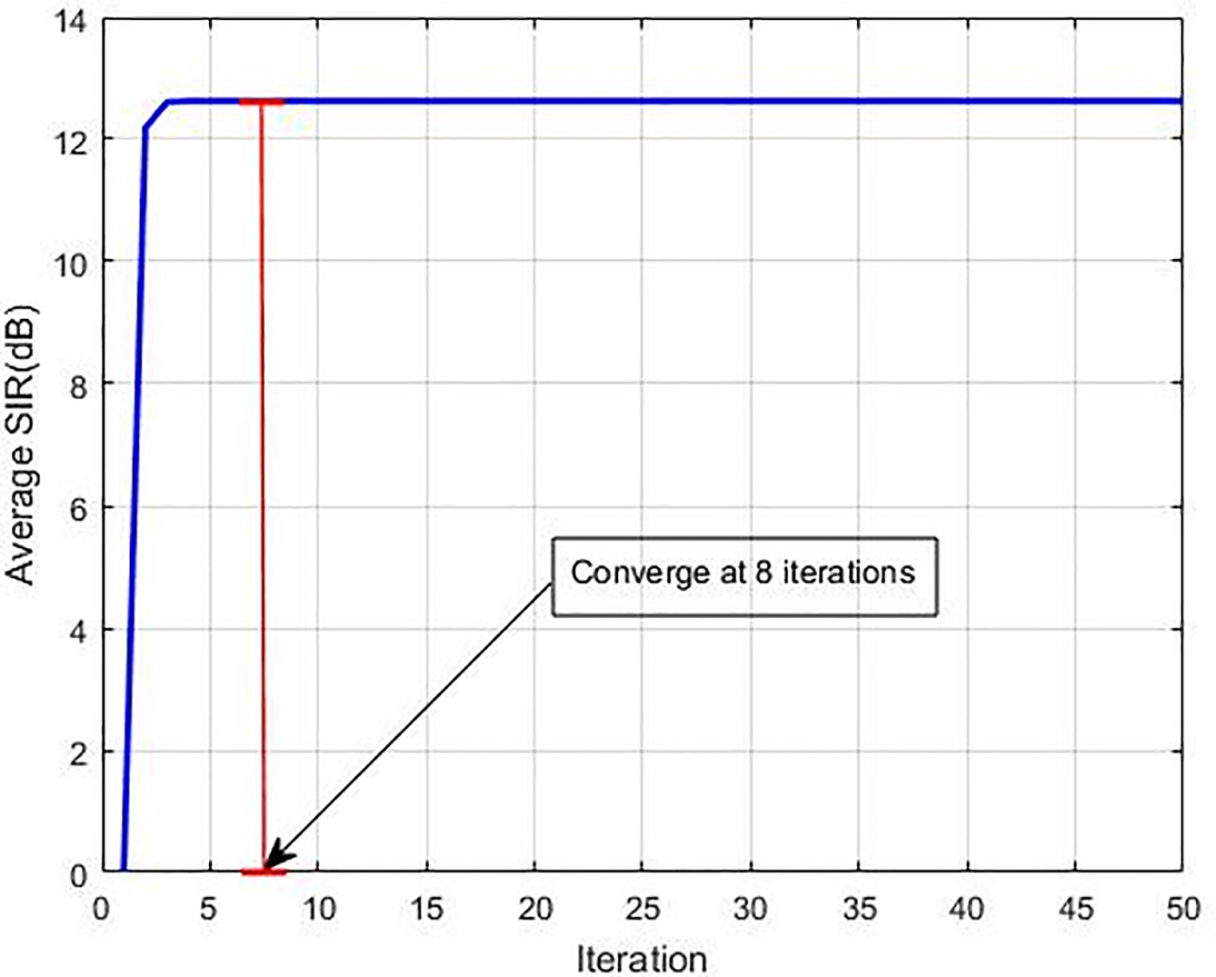
Convergence of average SIR in the proposed algorithm.

**Fig 11:**
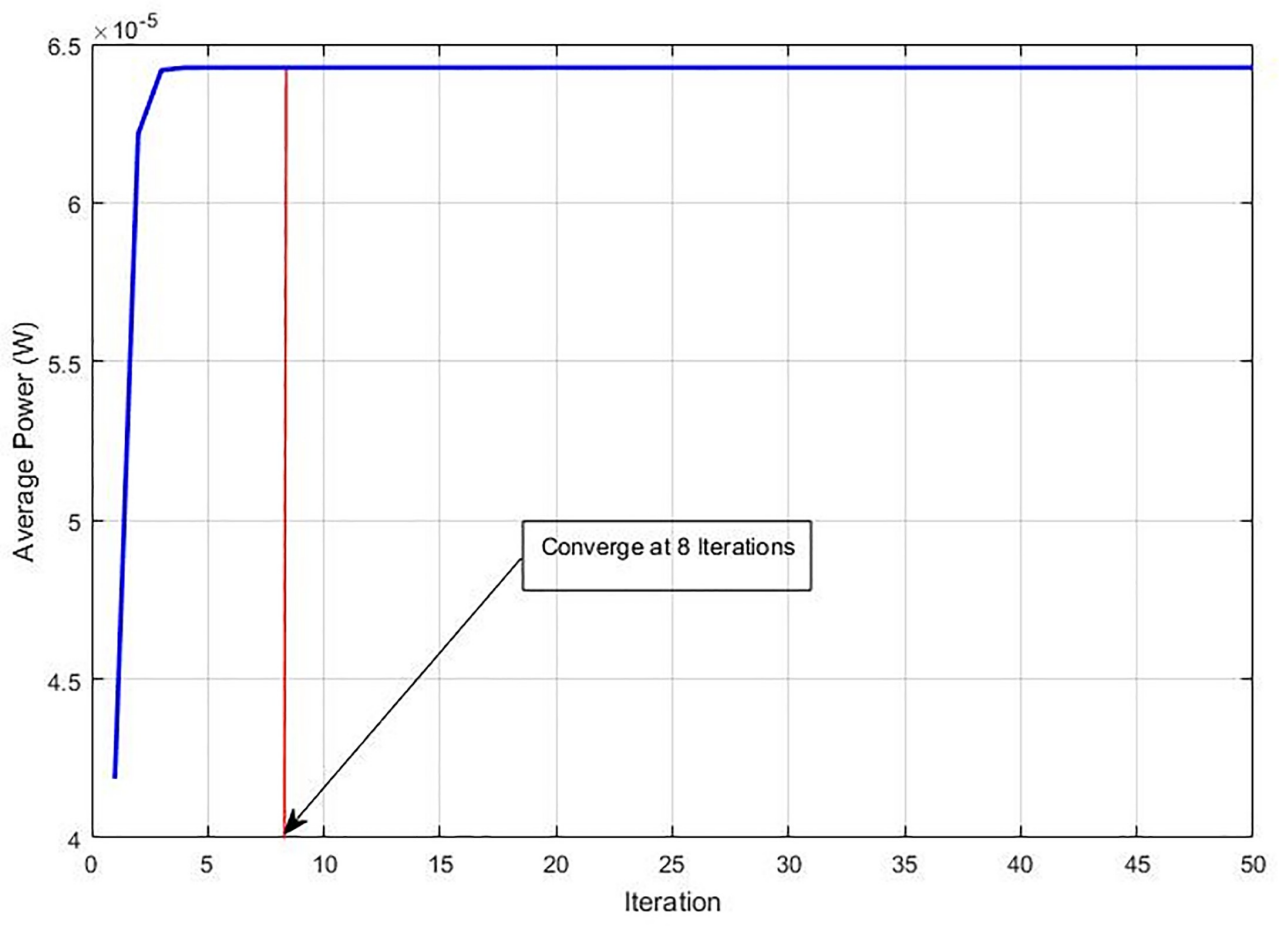
Convergence of average power in the proposed algorithm.

Additionally, we examine the average SIR and average power in comparison to the other methods to find out their convergence speed and decrease in the average power. In this experiment, the iteration time is shown on the horizontal axis that is required to achieve the Nash Equilibrium, while the average power and average SIR are shown on the vertical axis. It is displayed in Table 2 that almost all methods obtain the similar average SIR without any noteworthy variances but there is a difference in the convergence speed for every method. It is observed that our recommended GC-NPGP method can achieve Nash Equilibrium with just 8 iterations as displayed in Fig 10, while for NPG-MSFLA, NPGP-ESIA, R-NPGP, and EF-NPGP, it requires 333, 360, 323 and 133 iterations, respectively. Alternatively, Fig 11 displays the curve of average transmission power obtained by the recommended algorithm. Fig 11 reflects that the usage of average power of the recommended GC-NPGP method has a considerable decrease compared to other methods and that is the most important thing in implementing potential 5G wireless networks. We succeeded in decreasing the transmission power from Watt to micro-Watt as can be seen in Fig 11. The outcome attained from Fig 11 signifies interference’s extent gauged at the main system from the suggested GC-NPGP is considered the least against other algorithms; this attribute of the suggested GC-NPGP algorithm makes it apt for maximising the sharing of spectrum, and guarantees QoS in either systems. The convergence speed of the recommended algorithm is apparent from Fig 11, in which it can be seen that the recommended GC-NPGP method has the highest convergence speed compared to other methods as given in Table 3, shows power consumption average (in Watts) and the required iterations of all the methods.

**Table 3:** Average power and NE convergence comparison.

Algorithm	Average Power (W)	Number of Iterations
NPG-MSFLA [18]	0.2321	333
NPGP-ESIA [17]	0.2319	360
R-NPGP [16]	0.2287	323
EF-NPGP [15]	0.1926	133
GC-NPGP	6.4257e-05	8

## Conclusion

We have proposed a non-cooperative green communication technique in CRNs within wireless networks. The CR users’ QoS represent an effective utilisation through pricing and energy harvesting coefficient. By presenting utility functions and new price as an effective non-cooperative green transmission game is created and Nash Equilibrium’s uniqueness and its existence are confirmed mathematically. In this work, statistical outcomes suggest that the non-cooperative green transmission technique has two very important specifications such as lower power consumption and faster convergence speed simultaneously in comparison with the other methods found in the reviewed literature. Moreover, most of the nearby CR users in the method recommended by us can get better SIR than that of the other methods. The higher cost is only utilised to the most distant users that signify a more significant source of unwanted interference. The presented method provides better performance, wherein the CRN can share the additional licensed bands by staying under the limits of the interference temperature. The considerable decrease in the power consumption of the recommended method proves to be of the highest importance for application in cognitive radio in the 5G network domain. Further, we will consider an adaptation of the method to choose the best pricing values parameters *c*_*best*_ and *α*_*best*_, at the base station, these parameters can be employed to obtain a considerable improvement to be used in networks beyond the 5G technology.
