# Correction: Green communication for cognitive radio networks based on game and utility-pricing theories

**DOI:** 10.1371/journal.pone.0292778

**Published:** 2023-10-05

**Authors:** Fadhil Mukhlif, Kamarul Ariffin Bin Noordin, Omar B. Abdulghafoor, Tengku Faiz Tengku Mohmed Noor Izam

There are errors in the Funding section. The correct Funding statement is: The authors would like to acknowledge the Fundamental Research Grant Scheme (FRGS) from the Ministry of Higher Education, Grant no: FRGS/1/2018/TK04/UM/02/1 (FP091-2018A).
